# Assessment of Metagenomic Assembly Using Simulated Next Generation Sequencing Data

**DOI:** 10.1371/journal.pone.0031386

**Published:** 2012-02-23

**Authors:** Daniel R. Mende, Alison S. Waller, Shinichi Sunagawa, Aino I. Järvelin, Michelle M. Chan, Manimozhiyan Arumugam, Jeroen Raes, Peer Bork

**Affiliations:** 1 European Molecular Biology Laboratory, Heidelberg, Germany; 2 Computational and Systems Biology Program, Massachusetts Institute of Technology, Cambridge Massachusetts, United States of America; 3 VIB, Vrije Universiteit Brussel, Brussels, Belgium; 4 Max Delbrück Centre for Molecular Medicine, Berlin, Germany; Hospital for Sick Children, Canada

## Abstract

Due to the complexity of the protocols and a limited knowledge of the nature of microbial communities, simulating metagenomic sequences plays an important role in testing the performance of existing tools and data analysis methods with metagenomic data. We developed metagenomic read simulators with platform-specific (Sanger, pyrosequencing, Illumina) base-error models, and simulated metagenomes of differing community complexities. We first evaluated the effect of rigorous quality control on Illumina data. Although quality filtering removed a large proportion of the data, it greatly improved the accuracy and contig lengths of resulting assemblies. We then compared the quality-trimmed Illumina assemblies to those from Sanger and pyrosequencing. For the simple community (10 genomes) all sequencing technologies assembled a similar amount and accurately represented the expected functional composition. For the more complex community (100 genomes) Illumina produced the best assemblies and more correctly resembled the expected functional composition. For the most complex community (400 genomes) there was very little assembly of reads from any sequencing technology. However, due to the longer read length the Sanger reads still represented the overall functional composition reasonably well. We further examined the effect of scaffolding of contigs using paired-end Illumina reads. It dramatically increased contig lengths of the simple community and yielded minor improvements to the more complex communities. Although the increase in contig length was accompanied by increased chimericity, it resulted in more complete genes and a better characterization of the functional repertoire. The metagenomic simulators developed for this research are freely available.

## Introduction

The field of metagenomics examines the functional and phylogenetic composition of microbial communities in their natural habitats and allows access to the genomic content of the majority of organisms that are not easily cultivatable [1]. This is achieved through extraction of genomic DNA directly from environmental samples followed by sequencing, assembly and data analysis. Metagenomics has lead to the characterization of microbial communities in a variety of habitats on the earth: for example, the ocean [2]–[3], soil [4]–[5], hot springs [6] and acid-mine drainage ponds [7]–[8]. More recently the human microbiome, in particular the gastro intestinal tract [9]–[11], gained considerable attention and large-scale metagenomic initiatives now promise to characterize the microbiota in many different body sites with an ultimate goal of understanding human health and disease (e.g. [12]). The very first projects used Sanger sequencing, and even though Sanger sequencing is used less and less due to the advent of less expensive next generation sequencing, it still can reveal novel biological concepts [11]. In addition, reanalysis of Sanger sequencing data have led to a number of recent discoveries [13]–[15]. Yet, the currently two most prominent sequencing methods used for metagenomics are pyrosequencing [16]–[17] and most recently Illumina sequencing [10] enabling studies of a wide array of ecosystems, with the consequence of an exponential increase in environmental sequencing [18].

The initial steps in metagenomic data analysis involve the assembly of DNA sequence reads into contiguous consensus sequences (contigs), followed by prediction of genes. The protein-coding genes are then used to predict the functional repertoire encoded in the metagenomes and the phylogenetic composition can be estimated using a variety of methods [19]. Data analysis pipeline tools like SmashCommunity [20], MG-RAST [21], IMG/M [22] and Metarep [23], are complemented by numerous special purpose tools, and they all need to be validated. As there is no completely annotated metagenome available, simulations based on genomic data provide the currently only feasible way to get close to the truth. Indeed a number of simulations have already been performed in metagenomics. Mavromatis and colleagues [24] simulated metagenomic data by sampling sequencing reads from isolate genomes and then benchmarked assembly and annotation tools for Sanger-sequenced metagenomes. In addition, some simulator software has been developed that allows users to create metagenomes with desired properties: MetaSim [25], Grinder [26] and NGSfy [27].

Here we investigate the fidelity of metagenomic assemblies of next generation sequencing methods (pyrosequencing and Illumina) and compare these to classical Sanger sequencing as well as to previous results. To enable this, we developed two new metagenomic simulators iMESS (for Sanger and pyrosequencing) and iMESSi (for Illumina) that not only provide realistic sequencing reads, but also simulate errors and corresponding quality values based on actual metagenomic data. The simulated metagenomes were used to benchmark currently used assembly protocols. Due to the current uprise of Illumina sequencing in metagenomics, we also assessed the impact of quality control as well as the use of scaffolding in metagenomics. The simulators are freely available to allow the design of custom metagenomic data, and in order to allow researchers to benchmark new tools using these datasets the raw and assembled data are available at http://www.bork.embl.de/~mende/simulated_data/.

## Methods

### iMESS

iMESS is a metagenomic simulator for Sanger sequencing as well as pyrosequencing. Users can generate Metagenomes through an easy-to-use website at http://www.bork.embl.de/software/iMESS. First the user has to specify a desired community structure, by selecting the number of organisms and the shape of the rank abundance curve. Next the sequencing method and the amount of sequencing (number of reads) have to be specified. Using these parameters the simulator calculates how many reads of each organism's genome should be sequenced. iMESS then calls ReadSim (http://ab.inf.uni-tuebingen.de/software/readsim/) to generate reads with sequencing errors but without quality values. Quality value models were determined by obtaining quality values from actual metagenomic reads and fitting a function. For Sanger sequences quality models were generated for 3 different data sets: JGI [7] [28], TIGR [9], JAP [29]. For pyrosequencing one model was determined based on reads from a real dataset (unpublished) with a 250 bp median length. Read sequences and quality values are written to a .fasta and a .qual file. For more details please refer to the iMESS manual online.

### iMESS_Illumina

iMESSi is a metagenomic simulator for the Illumina sequencing technology. It can be downloaded at: http://sourceforge.net/projects/cmessi/. Similar to iMESS the user first specifies what kind of community should be simulated. The user also has to specify a number of other parameters including the total number of inserts, the read length, the insert size and standard deviation of the read length. The actual number of inserts sampled from each genome is calculated in a similar fashion as done in Metasim [25]. To generate realistic quality values we obtained quality values from the MetaHIT gene catalog dataset [10] and clustered the runs by quality values using Euclidean distances. This resulted in 3 different clusters for 75 bp reads and one for 44 bp reads. To simulate errors within the reads the quality values are then mapped to a ‘read’ extracted from a reference genome and then random errors were generated at the probability as defined by the equation below.

Q = −10 log_10_ (P/(1−P)), where Q is the Phred quality score and P is the error probability [30].

The 4 error models for 75 bp reads show large differences in average error. The error models are available at: http://sourceforge.net/projects/cmessi/, but users can easily generate their own error models by extracting the quality values from any Illumina sequencing run and converting them to Sanger scale Phred scores in .qual format. This enables users to generate realistic data for their sequencing machine and protocol and enables simulations of Illumina reads from any sequencing read length used by Illumina sequencing. The sequences and their assigned quality values are returned as fastq formatted files [31].

### Simulated Metagenomes

Metagenomic datasets were simulated for Sanger sequencing, pyrosequencing, and Illumina sequencing. For each sequencing technology, three metagenomes were simulated to mimic different community complexities (10, 100 and 400 genomes). (Table 1, Table S1, S2, S3). We generated metagenomes of the three sequencing platform at different sequencing depths in order to account for the price difference between the three sequencing technologies and the usual sequencing effort for metagenomic projects using each technology. Thus, about 15 times more base pairs were generated for Illumina than for pyrosequencing, to reflect the lower cost associated with Illumina sequencing [32], and similarly 1.3 times more for pyrosequencing than for Sanger. The datasets were assembled and analyzed using SmashCommunity (pyrosequencing and Sanger) or a pipeline using freely available tools (i.e fastx toolkit [33], SOAPdenovo [34] and parts of the SmashCommunity pipline) (Illumina).

**Table 1:** Simulated Raw Data for each Metagenome.

Simulated Metagenome	MG1	MG2	MG3	MG4	MG5	MG6	MG7	MG8	MG9
Sequencing technology	Illumina			Sanger			pyrosequencing		
**Number of genomes**	10	100	400	10	100	400	10	100	400
**Number of reads (Million)**	53.33	53.33	53.33	0.25	0.25	0.25	1.00	1.00	1.00
**Amount sequence (Mb)**	4000	4000	4000	200	200	200	255	255	255
**Average read length (bp)**	75	75	75	800	800	800	255	255	255

For each sequencing technology (Illumina, Sanger, pyrosequencing), three different metagenomes were simulated for different community complexities (10 genomes, 100 genomes, 400 genomes). The amount of sequence generated for each sequencing technology was based on the current price for each technology as well as the usual amount generated. Reads and quality values for Illumina were generated using the freely available simulator iMESSi, and reads and quality values for Sanger and pyrosequencing were generated using iMESS which is available through an easy-to-use web interface.

### Quality Control

Sanger and pyrosequencing reads were quality trimmed using lucy [35] and the lucyTrim.pl script from OCTUPUS (http://octupus.sourceforge.net). Illumina reads were quality trimmed and filtered using the procedure described in [11]. Specifically, the 5′-ends of the reads were trimmed so that the abundance of each base (A,C,T,G) per position was within 2 standard deviations of the average across all cycles. Then, all bases with a quality score less than 20 were trimmed off the 3′-ends. Lastly, all reads that were shorter than 35 bases or had a median quality score below 20 were removed.

### Mapping of Illumina Reads to Original Genomes

Trimmed and untrimmed reads were mapped to the original genomes with MOSAIK 1.1.0021 (http://bioinformatics.bc.edu/marthlab/Mosaik) using the following parameters: “-a all -m all -hs 15 -mm 2 -act 35”. This software was also used to calculate the coverage of the genomes.

### Assemblies

All simulated metagenomic datasets using Sanger and pyrosequencing technologies were assembled using SmashCommunity [20]. The Sanger sequencing data was assembled using Arachne v3.1 [36] with SmashCommunity standard parameters. Pyrosequencing datasets were assembled using Celera [37] assembler with SmashCommunity standard parameters.

The Illumina datasets were assembled using SOAPdenovo 1.05 [33] using following parameters: “-K 23 -L 70 -M 3 -u -R -F”. To assess the effect of quality filtering in metagenomic data analysis of Illumina data, we assembled the datasets with and without quality filtering, as described above.

To determine which read was incorporated into which contig we used this information provided by SmashCommunity for all datasets processed with this tool. In order to get this information for the Illumina datasets we mapped the reads against the contigs using SOAPaligner 2.20 (Parameters: “-r 0 -v 2 -M 2”) [38].

### Measures of Chimerism

Chimeric contigs are those contigs that combine reads originating from more than one genome. This definition was originally based on assemblies of Sanger reads. In contrast to assemblies of Sanger reads, in assemblies of Illumina data reads can be assigned to more than one contig as an entire read may be identical (or nearly identical) to two reference genomes. Therefore to adjust the definition of a Chimeric contig to Illumina data, contigs were only considered to be chimeric if they contained uniquely-mapping reads that originate from more than one genome. Uniquely-mapping reads are those that are only mapped to one contig, as opposed to being mapped to multiple contigs. For all chimeric contigs we calculated the degree of chimericity as described in [24]. Specifically, the degree of chimericity is the ratio of the number of reads that do not originate from the species which makes up most of the reads in the contig over the total number of reads in that contig.

### Contig Score

In order to determine how accurately contigs represent the corresponding genomes, we defined the Contig Score. To calculate this we used BLASTN to map contigs to the original genomes. We then extracted the percent identity for the best HSP as well as the percent of each contig covered by its HSP. The Contig Score was then calculated by multiplying these two values and normalized to be in a range from 0 to 100.

### Functional Annotation and Analysis

Functional annotation and analysis was done using SmashCommunity [20]. Briefly, gene prediction was performed using MetaGeneMark [39], and then the protein translations of the predicted genes were assigned to a COG (Cluster of Orthologous Group) by performing a BLASTP against the eggNOG2 database (single best hit, bit score >60) [40]. The abundance of each COG in each metagenome was determined using scripts in SmashCommunity. The abundances were normalized to produce probability distributions. To determine the similarity or difference between the COG abundance distributions, Principal Coordinate Analysis (PCoA) was performed using a distance metric related to Jensen-Shannon Divergence (JSD) [11]. For a complementary ordination analysis, Principal Component Analysis (PCA) was performed using the COG abundance distribution matrix using R [41]. In addition, the abundance of each COG, as determined from the metagenomic assembly and annotation, was plotted against the abundance of each COG as expected from the input genomes and Pearson correlation coefficients were determined.

## Results

### Importance of Quality Control for Illumina Data

**Figure 1:**
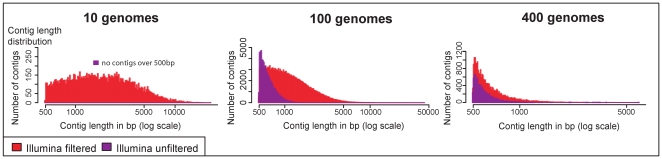
Comparison of Assemblies of Illumina Data with and without Quality Control. Contig length histograms illustrate the number of contigs within a certain size fraction for assemblies of Illumina reads with quality filtering (red) and without quality filtering (purple). Contig lengths were compared for assemblies of different community complexities (10 genomes, 100 genomes, 400 genomes). Only contigs greater than 500 bp are shown, the x-axis is log scale. There was a strong improvement in the assemblies with pre-assembly quality control of the reads.

Both metagenomic simulators presented here generate sequences and corresponding quality values that were modeled on actual metagenomic data. Quality values were originally developed for Sanger sequencing to estimate the accuracy of each base call [42]. Most assemblers for Sanger and pyrosequencing use quality values as part of the assembly process (e.g. Phrap (http://www.phrap.org/), Arachne [36], JAZZ [43], Celera [37], Newbler [44]). However, the most commonly used assemblers for Illumina data (SOAPdenovo [33], Velvet [45], SSAKE [46] do not use quality values for assembly. Thus there is no standard treatment for poor quality bases of Illumina reads. To evaluate the impact of quality-based pre-processing of reads, the 3 Illumina metagenomic datasets were assembled with and without quality control. We chose quality control which included trimming the 5′-end based on base frequency distributions, the 3′-end based on quality scores, and then removal of reads based on median quality scores and minimum length. This is more rigorous than the quality control performed with the first published Illumina metagenomic data set [10]. Although 13–16% of the reads and 24–27% of the base pairs were removed by quality trimming, the accuracy of the data was greatly improved (Table 2). To assess how well the reads represent the genomic sequences present in the metagenome, the reads were mapped back to the source genomes allowing for a maximum of 2 mismatches. Even though the total number of reads was lower in the trimmed dataset, the total number of reads that mapped to the original genomes doubled. The quality trimming of the reads also produced a strong improvement in the assemblies (Figure 1, Table 2). Notably, for the 10 species metagenome, prior to quality trimming no contigs longer than 500 base pairs (bp) were obtained, while after trimming 13799 contigs longer than 500 bp were assembled with an average length of 2332 bp. For the 100 genomes metagenome the number of contigs longer than 500 bp increased by almost 3-fold and the N50 more than doubled. While, for the 400 genomes metagenome improvements to the assembly due to quality filtering were more modest. Increasing the number of contigs longer than 500 base pairs will strengthen the ability to predict and annotate protein-coding genes by both using homology- and neighborhood-based [47] methods. Assembly of the trimmed dataset clearly outperforms the assembly of the untrimmed dataset demonstrating that stringent quality control as performed here should be used for real metagenomic sequencing data in order to enhance results.

**Table 2:** Comparison of Reads and Assemblies for Illumina Data with and without Quality Control.

Metagenome	10 genomes			100 genomes			400 genomes		
	raw	qualitly filtered	fold change	raw	quality filtered	fold change	raw	quality filtered	fold change
**raw data**									
**Bases (Mbp)**	4000	2908.95	0.73	4000	3031.64	0.76	4000	3031.68	0.76
**Reads (Million)**	53.33	44.99	0.84	53.33	46.47	0.87	53.33	46.47	0.87
**Average Read Length**	75.00	64.66	0.86	75.00	65.24	0.87	75.00	65.24	0.87
**accuracy of raw data**									
**Mapped Reads (max. 2 MM)**	23.36	42.99	1.84	25.48	44.56	1.75	25.48	44.56	1.75
**% mapped of total**	43.80	95.55	2.18	47.78	95.90	2.01	47.77	95.89	2.01
**% of ref genome covered**	100	100	1.00	98.2	98.7	1.01	75.3	87.8	1.17
**contig lengths in assembly**									
**Number of Contigs >500 bp**	0	13799	n/a	65813	183528	2.79	10154	23794	2.34
**Average Contig Length**	n/a	2332	n/a	647	1291	2.00	589	679	1.15
**N50**	n/a	3240	n/a	629	1496	2.38	567	635	1.12
**Longest Contig**	354	22725	n/a	2448	43111	17.61	1601	5854	3.66

The table is divided into three sections. The upper section describes the raw data before and after quality control. The second, describes the accuracy of the data as determined by mapping the reads back to the reference genomes (allowing for 2 mismatches). The last section compares the length of the contigs that result from assembly of the raw and trimmed data. N50 is defined as the length N for which 50% of all bases are represented in fragments of length L<N. The degree of chimericity is the ratio of the number of reads that do not originate from the species which makes up most of the reads in the contig over the total number of reads in that contig. The Contig Score represents the percent identity between a contig and its corresponding reference genome. The percent identity between the two (from BLAST) is multiplied by the percent of each contig covered by its HSP and normalized to be in a range from 0 to 100.

### Comparison of Assemblies from Different Sequencing Methods

We used different assembly programs for reads generated by each sequencing technology in order to account for the differences between them. Therefore, the following is a comparison between assemblies produced from different sequencing methods, along with a chosen pipeline of assembly software with specified parameters. We used parameters which are optimized for metagenomic assembly as well as for each technology and that were used in previous studies [11] [10].

**Figure 2:**
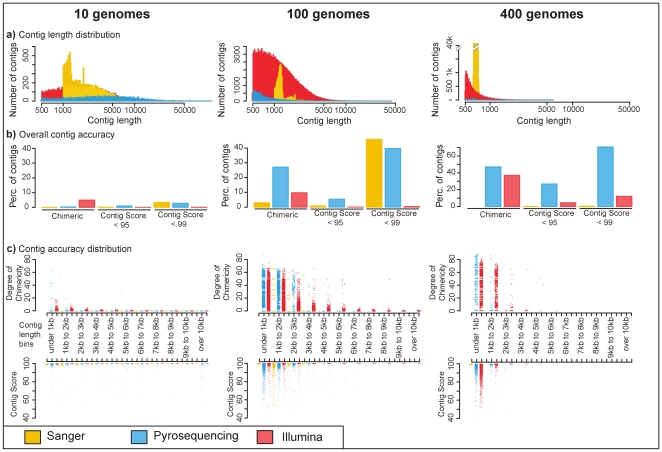
Comparison of assemblies from different sequencing technologies. a) **Contig Length Distribution.** Histograms of the contig lengths illustrate the number of contigs within a certain size fraction for assemblies of Illumina reads with quality filtering (red), Sanger sequenced reads (yellow) and reads from pyrosequencing (blue). Only contigs greater than 500 bp are shown, the x-axis is log scale. Assemblies were generated for different community complexities (10 genomes, 100 genomes, 400 genomes). b) **Overall Accuracy of the Contigs.** The overall accuracy of the contigs is summarized using different measures of chimericity. Bars to the left illustrate the percentage of all of the contigs that are chimeric, bars in the middle show the percentage of all of the contigs that have a Contig Score less than 95%, and to the right contigs that have a Contig Score less than 99%. Contig Score represents the percent identity between the contig and the derived reference genome. Contigs from Illumina reads are red, contigs from Sanger reads are yellow and contigs from pyrosequencing are blue. In general there was a slightly higher proportion of Illumina contigs that were chimeric, however they had higher contig scores. c) **Contig Accuracy across Contig Lengths.** These combined strip plots show the degree of chimericity (upper plot) and contig score (lower plot) for each contig in the assemblies, each dot represents one contig. They are grouped into size bins. The degree of chimericity is the proportion of reads in a contig that are derived from the ‘wrong’ genome and thus make the contig chimeric. Contig Score represents the percent identity between the contig and the derived reference genome. Again contigs from Illumina assemblies are in red, from Sanger assemblies are in yellow and from pyrosequencing assemblies are in blue. For all sequencing technologies and communities, longer contigs are more accurate.

A comparison of the assemblies shows that contig size length distributions differ depending on the community complexity and the sequencing technology (Figure 2, Table 3). For simple communities (10 genomes) all sequencing platforms produced a similar total sum of contig lengths, but differed in the distribution of contig lengths. Although pyrosequencing had the longest N50 (N50 is defined as the length N for which 50% of all bases are represented in fragments of length L<N) [48], Sanger sequencing produced the largest number of contigs greater than 500 bp. For more complex communities (100 genomes), Illumina reads resulted in by far the best assembly with 8 times the number of contigs assembled than as for the Sanger sequences (the next best), the largest proportion of long contigs, and over 6 times more genes with functional annotations. For the most complex community (400 genomes) there was very little assembly using any technology. Although there was no assembly of Sanger reads into contigs, the Sanger reads still represent the best ‘assembly’ with 10 times more fragments over 500 bp, than the Illumina assembly and over 10 times more genes with functional annotation. These differences can be attributed to the differences in read length and especially sequencing depth, but both parameters are intrinsic to the different sequencing technologies., as the cost of the sequencing technology is directly related to the sequencing depth. All of the sequencing technologies perform comparably when the coverage per organism is relatively high as in the 10 genomes metagenome. But for the more complex communities (100 genomes) Illumina performs better due to the greater sequencing depth achieved. However, for the metagenomes with 400 genomes even the sequencing depth achieved with Illumina does not make up for the lower coverage of each genome and Sanger performs well due to the long read lengths.

**Table 3:** Comparison of Assemblies from Reads of Different Sequencing Technologies.

Number of Genomes	10			100			400		
Sequencing	Illumina	Sanger	pyro	Illumina	Sanger	pyro	Illumina	Sanger	pyro
**contig lengths** 1									
**Number of Contigs >500 bp**	13799	**15368**	6046	**183528**	21977	14035	23634	**249989**	1151
**Sum of Contig Lengths (Mbp)**	**32.18**	**34.88**	**33.26**	**236.91**	28.33	11.71	16.05	**167.89**	0.81
**N50 (bp)**	3240	2693	**9198**	**1496**	1221	809	636	671	640
**Longest Contig (kb)**	22.73	13.70	**119.83**	**43.11**	4.83	7.26	5.85	0.77	2.81
**contig accuracy**									
**% of contigs that are chimeric**	4.98	**0.01**	0.36	9.79	**2.98**	27.10	37.08	**0** 2	47.15
**Average degree of chimericity (%)**	1.56	**38.04**	15.28	18.25	**44.56**	35.67	41.50	0^2^	47.89
**% of contigs with ContigScore <95**	**0.03**	0.24	1.62	0.11	0.84	**5.50**	4.51	0.38	**26.80**
**functional annotation**									
**Number of genes**	40173	52550	40201	359969	61839	26369	33449	413779	**1587**
**Number of complete genes**	21276	28841	31628	106024	30899	9228	3267	102121	**542**
**Number of genes with OG annotation**	35713	42878	35399	305217	48922	20992	27118	293047	**1002**

1 - for Sanger reads there were no contigs assembled from combining more than one read, therefore the term contigs represents Sanger reads that were longer than 500 bp.

2 – as there were no contigs assembled from more than one read, then there cannot be any ‘chimeric’ contigs.

This table compares assembly statistics for assemblies of reads from quality-trimmed Illumina reads, Sanger reads, and pyrosequencing reads (quality trimming was performed by assembly software). The upper portion of the table compares different statistics related to contig lengths, and the lower portion compares statistics related to the accuracy of the contigs. N50 is defined as the length N for which 50% of all bases are represented in fragments of length L<N. The degree of chimericity is the ratio of the number of reads that do not originate from the species which makes up most of the reads in the contig over the total number of reads in that contig. The Contig Score represents the percent identity between a contig and its corresponding reference genome. The percent identity between the two (from BLAST) is multiplied by the percent of each contig covered by its HSP and normalized to be in a range from 0 to 100.

The definition of a chimeric contig arose from analysis of Sanger assemblies and describes a contig that combines reads originating from more than one genome [24]. In this case, reads that originate from two different genomes may be combined into one contig based on a short region of homology between the two, while the majority of the contig would match one of the reference genomes better than the other. However for Illumina reads the definition of a chimeric contig is not as clear. As Illumina reads are so short, an entire read may be identical (or nearly) to two reference genomes. In addition, most Illumina assembly software allows reads to be assigned to more than one contig. Thus, for Illumina data we defined a contig as chimeric if it contains uniquely-mapped reads that originate from more than one genome; where uniquely mapped reads are those that are only mapped to one contig, as opposed to being mapped to multiple contigs. Moreover, in order to assess the accuracy of a contig without using the concept of chimericity, which may not be so informative for Illumina data, we defined the term ‘contig score’ to represent the sequence identity between a contig and its corresponding genome. The contig score can vary between 0 and 100, with 100 being the best value.

The percentage of chimeric contigs was lowest in Sanger sequencing, while pyrosequencing and Illumina had a much higher percentage of chimeric contigs (Table 2, Figure 2). However, the degree of chimericity (percentage of reads from “wrong” genomes [24]) was on average higher for Sanger sequencing than the other sequencing technologies, with the effect being more pronounced in the least complex community, and almost negligible in the most complex community. The degree of chimericity was clearly dependent on the contig length for all sequencing methods, with shorter contigs having a much higher degree of chimericity. The percentage of chimeric contigs and the degree of chimericity both increase with increasing community complexity. This shows that some contig accuracy characteristics known from Sanger sequencing [24] also hold true for next generation sequencing methods.

By comparing the contig scores to the chimericity analysis, it is clear that chimeric contigs can still resemble the original genomes (Figure 2, Panel B). This is especially true for Illumina contigs that had overall better contig scores than other sequencing method for all community complexities. Conversely, pyrosequencing produced assemblies with the lowest contig scores. In agreement with the relationship between contig length and chimericity, we also found a proportional relationship between contig length and the contig score in most cases, delivering additional evidence that longer contigs are indeed more reliable. However, for the 10 genome community the trend was not as clear for the contigs from pyrosequencing, and a number of long contigs had a low contig score.

### The use of Scaffolds and Scaftigs in Illumina Assemblies

The assembly of reads into contigs usually does not lead to completely assembled genomes, hence scaffolding is used to combine contigs and place them within context of their genomic location [49]. Scaffolds are constructed by linking contigs using information from paired end reads. During this process a number of unknown bases, or gaps, are usually found between the sequences of the linked contigs. Some scaffolding tools try to fill this gapped-region with unused reads. Unknown bases that remain between the contigs in the scaffold will be represented by Ns. To use the information obtained by scaffolding, scaftigs can be constructed by extracting the contiguous sequences that lack unknown bases (Ns). Scaffolding is especially useful when assembling short reads generated using next generation sequencing technologies since repeats are harder to resolve in this case. The main advantage of scaftigs over contigs is an increase in fragment lengths and scaftigs have been proven to be useful in metagenomic data analysis [10].

**Figure 3:**
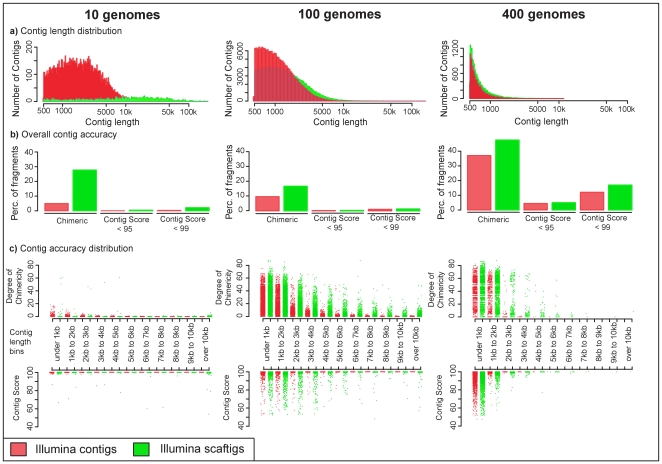
Comparison of assemblies of Illumina contigs and Illumina scaftigs. Scaffolds are constructed by linking contigs using information from paired end reads, during this process a number of unknown bases are usually found between the sequences of the linked contigs. To use the information obtained by scaffolding, Scaftigs can be constructed by extracting the contiguous sequences that lack unknown bases (Ns). a) **Contig Length Distribution.** Histograms of the contig lengths illustrate the number of contigs within a certain size fraction for assemblies of Illumina contigs (red) and Illumina scaftigs (light blue). Only contigs greater than 500 bp are shown, the x-axis is log scale. Assemblies were generated for different community complexities (10 genomes, 100 genomes, 400 genomes). b) **Overall Accuracy of the Contigs.** The overall accuracy of the contigs is summarized using different measures of chimericity. Bars to the left illustrate the percentage of all of the contigs that are chimeric, bars in the middle show the percentage of all of the contigs that have a Contig Score less than 95%, and to the right contigs that have a Contig Score less than 99%. Contig Score represents the percent identity between the contig and the derived reference genome. Illumina contigs are in red Illumina scaftigs are blue. c) **Contig Accuracy across Contig Lengths.** These combined strip plots show the degree of chimericity (upper plot) and contig score (lower plot) for each contig in the assemblies, each dot represents one contig. They are grouped into size bins. The degree of chimericity is the proportion of reads in a contig that are derived from the ‘wrong’ genome and thus make the contig chimeric. Contig Score represents the percent identity between the contig and the derived reference genome. Again Illumina contigs are in red and Illumina scaftigs are in blue.

We used simulated Illumina data to survey the effect of scaffolding on assemblies of different communities. Fragment lengths increase with scaffolding. This is most pronounced in the low complexity metagenomes (10 genomes) where the N50 increases 10-fold from 3240 to 35893, while there is hardly a difference in the high complexity (400 genomes) dataset (N50 improves from 631 to 690) (Table 4, Figure 3). Although the use of scaffolds increases contig lengths, a larger proportion of scaftigs were chimeric than contigs in simulations of all community complexities. The degree of chimercity was also higher in scaftigs than in contigs for all length bins (Figure 3). While the effect of scaffolding seems to be detrimental when looking at chimericity, the effect is small when comparing the actual sequence of scaftigs and contigs to the original genomes. The percentage of sequences having a contig score below 95 slightly increases for all 3 community complexities; however, the median contig score was very similar for scaftigs and contigs (Table 4). In conclusion, scaffolding of Illumina data represents a tradeoff between increased fragment lengths and accuracy, and therefore might be more useful when mapping fragments for function assignment purposes but less so when sequence identify is used to quantify the distance to reference genomes.

**Table 4:** Comparisons of Illumina Assemblies of Contigs and Scaftigs.

Number of Genomes	10			100			400		
Sequencing	Illumina contigs	Illumina scaftigs	fold change	Illumina contigs	Illumina scaftigs	fold change	Illumina contigs	Illumina scaftigs	fold change
**contig lengths**									
**Number of Contigs >500 bp**	13799	1900	0.14	183528	152230	0.83	23634	32496	1.37
**Sum of Contig Length (Mbp)**	32.18	33.34	1.04	236.91	33.34	0.14	16.05	24.19	1.51
**N50 (bp)**	**3240**	**35893**	**11.08**	1496	2200	1.47	636	690	1.08
**Longest Contig (kb)**	**22.73**	**195.65**	**8.61**	**43.11**	**573.10**	**13.29**	**5.85**	**11.70**	**2.00**
**contig accuracy**									
**% of contigs that are chimeric**	**4.98**	**27.74**	**5.57**	9.79	16.82	1.72	37.08	41.50	1.12
**Average degree of chimericity (%)**	1.56	1.21	0.78	18.25	19.03	1.04	41.50	42.96	1.04
**% of contigs with ContigScore <95**	0.03	2.05	70.81	0.11	0.42	3.82	4.51	5.03	1.11
**functional annotation**									
**Number of predicted genes**	40173	32834	0.82	359969	358473	1.00	33449	47667	1.43
**Number of complete genes**	21276	30690	1.44	106024	144364	1.36	3267	6000	1.84
**Number of genes with OG annotation**	35713	29827	0.84	305217	305823	1.00	27118	39235	1.45

This table compares assembly statistics for assemblies of Illumina data that result in contigs and those for which scaftigs were created. N50 is defined as the length N for which 50% of all bases are represented in fragments of length L<N. The degree of chimericity is the ratio of the number of reads that do not originate from the species which makes up most of the reads in the contig over the total number of reads in that contig. The Contig Score represents the percent identity between a contig and its corresponding reference genome. The percent identity between the two (from BLAST) is multiplied by the percent of each contig covered by its HSP and normalized to be in a range from 0 to 100.

### Functional Composition of Simulated Metagenomes

**Figure 4:**
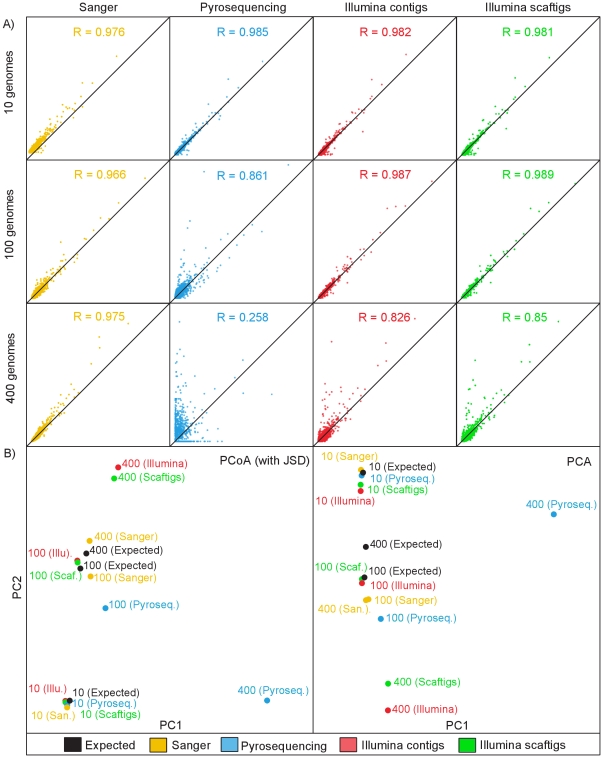
Comparison of the functional repertoire of the metagenomes to each other and to the expected. a) **Correlations between expected and actual COG abundance.** Dotplots compare the expected and actual abundance for each COG, with the x-axis displaying the COG abundances as expected from the input genomes and the y-axis displaying the COG abundances as determined from assembly and annotation of the simulated metagenomes. The black line shows the 1∶1 correlation. The Pearson correlation coefficients are displayed for each dataset. b) **Principal Coordinate Analysis (PCoA) and Principal Component Analysis (PCA).** The COG abundance profiles were compared to each other using Jensen-Shannon divergence and the distance matrix was then analyzed plotted using PCoA. The COG abundance profiles were analyzed plotted using PCA. The dots are colored by sequencing method: Illumina contigs (red), Illumina scaftigs (light blue), Sanger (yellow) and pyrosequencing (blue).

To determine if better assembly parameters such as longer contigs result in assemblies that more accurately represent the functional composition of the community, we compared the functional content of the metagenomes to the functional repertoire expected from the input genomes. For the 10 and 100 genome metagenomes, where the genomes had higher coverage, the actual COG abundances determined from the assemblies correlated well with the expected COG abundances (Figure 4A). For the more complex communities (100 and 400 genomes) the Illumina scaftigs had slightly better correlations than the Illumina contigs. This indicates that the positive impact of increased fragment length outweighs the minor increases in the number of assembly errors. In addition we performed principal component analysis and principal coordinate analysis of JSD distances to determine how the use of different technologies might affect functional ordination analyses. Both ordination analyses showed that for all sequencing technologies the low complexity metagenomes (10 genomes) were similar in functional content to each other as well as to the expected (Figure 4B). For the medium complexity metagenomes (100 genomes), the Illumina reads produced assemblies that were very similar to expected, with the Sanger assembly also being close and the pyrosequencing assembly being the most different from expected. For the high complexity community (400 genomes) the Illumina and pyrosequencing metagenomes appear to be quite divergent from the expected functional content. And the Sanger reads provided the best representation of the functional content with the Sanger assembly appearing relatively similar to expected in the PCoA. One of the main reasons that pyrosequencing could not accurately represent the overall functional composition was the lower number of genes that were annotated (Table 3). In addition, for all metagenomes the Illumina contigs and Illumina scaftigs had very similar functional compositions. Overall, the functional analysis shows that better assemblies (eg. more complete genes) do actually result in better functional characterization of a metagenome.

## Discussion

The first step in any metagenomic data analysis should be raw data treatment. This includes quality control and removal of contamination (eg. human contamination in metagenomic studies of the human gut). Tools for NGS quality control like the FASTX toolkit [33], SolexaQA [50] or PrinSeq [51] are readily available. However, there is currently no standard protocol for how the quality values should be used in read pre-processing. Our results show the importance of good quality control as the Illumina assemblies greatly improved after rigorous quality filtering and trimming.

After this initial step there are a number of analyses that one can perform, but to decrease the complexity of the data, the quality controlled reads are usually assembled into contigs. Our comparison of assemblies from different sequencing technologies reveals that each assembly has different characteristics depending on community composition and sequencing technology. The different sequencing technologies performed similarly for the low complexity community. For the more complex community (100 genomes), the Illumina sequences assembled a much greater length of contigs resulting in more complete genes. This is due to the greater sequencing depth achieved as the price per base is much less for Illumina sequencing than pyrosequencing or Sanger. For the most complex community none of the sequencing technologies assembled many reads into contigs. However, due to their length the Sanger reads still had many sequences greater than 500 bp. Earlier metagenomic simulation studies focused on the chimericity of contigs concluding that currently used assemblers need to improve to be useful for metagenomics [24]. However, this term that originated from Sanger sequencing may not be as applicable to IIlumina data. This is because Illumina reads are so short and may actually represent regions that are identical between two organisms, and because assembly of Illumina reads often results in reads being assigned to more than one contigs. We therefore need to asses new ways to determine the accuracy of Illumina assemblies. Accordingly, we defined the term ‘Contig Score’ to quantify the divergence of the contigs from the original genomes. Our results show that for all sequencing technologies and community complexities the vast majority of the contigs diverge by less than 5% from the original genomes. The Illumina dataset excelled using this measure showing the usefulness of Illumina sequencing data in metagenomics. The reliability of most contigs is reflected by the fact that the functional repertoire of the low and medium complexity metagenomes accurately represents the expected functional repertoire. This is also because the amount of sequence produced allowed for all of the sequencing technologies to provide enough coverage of each genome. For the most complex community, where there was low coverage of each genome, assemblies from Illumina and pyrosequencing failed to represent the expected functional composition of the metagenomes, as there were very few complete genes annotated. However, the Sanger reads approximated the expected functional composition reasonably well as the length of the reads allowed for accurate functional annotation. For the pyrosequencing simulations 250 bp was used as the average read length. Currently, the GS FLX Titanium system can deliver reads as long as 400 bp, this would probably improve the assemblies. The ability of all NGS sequencing technologies to fully capture the functional repertoire of complex communities will also improve as technology developments might lower prices allowing for deeper sequencing.

By using paired end information available in most NGS technologies, the fragment length of an assembly can be increased by gap-filling and scaffolding of contigs. Our results show that scaffolding is a good way to increase fragment lengths. And although scaffolding increased chimeras and decreased the Contig Score, the functional profiles of the metagenomes derived from contigs and those derived from scaftigs were virtually indistinguishable, with the COG abundance profiles of the scaftig-metagenomes correlating slightly better with the expected.

Currently, Illumina sequencing technology can produce the greatest yield at the lowest price [32], but as of now has not been extensively used for metagenomics. Our study of simulated metagenomes shows that Illumina data can be used to obtain assemblies that, for the low and medium complexity metagenomes in this study, are superior to those from pyrosequencing and Sanger sequencing, provided a rigorous quality control of reads prior to assembly. However, the assembly performance is coupled to the underlying community structure, and thus simulations will aid in choosing the optimal sequencing technology for a microbiome of interest. In addition, the results are highly dependent on the sequencing depth and read lengths which are increasing for next-generation sequencing technologies, thus it is likely that they may perform even better for more complex metagenomes in the future.

## Supporting Information

Table S1 **Genomes Used in the Low Complexity Metagenome and Estimated Coverage (10 genomes).**
(XLS)

Table S2 **Genomes Used in the Medium Complexity Metagenome and Estimated Coverage (100 genomes).**
(XLS)

Table S3 **Genomes Used in the High Complexity Metagenome and Estimated Coverage (400 genomes).**
(XLS)
