# Correction: Assessment of Metagenomic Assembly Using Simulated Next Generation Sequencing Data

**DOI:** 10.1371/journal.pone.0114063

**Published:** 2014-11-13

**Authors:** The PLOS ONE Staff

The first sentence of the second paragraph of the “Assemblies” subsection of the Methods should have cited reference 34 instead of 33. The correct sentence should read: The Illumina datasets were assembled using SOAPdenovo 1.05 [34] using following parameters: ‘‘-K 23 -L 70 -M 3 -u -R -F’’.

The fourth sentence of the first paragraph of the “Importance of Quality Control for Illumina Data” subsection of the Results should have cited reference 34 instead of 33. The correct sentence should read: However, the most commonly used assemblers for Illumina data (SOAPdenovo [34], Velvet [45], SSAKE [46] do not use quality values for assembly.
